# Whole Cell Imprinting in Sol-Gel Thin Films for Bacterial Recognition in Liquids: Macromolecular Fingerprinting

**DOI:** 10.3390/ijms11041236

**Published:** 2010-03-24

**Authors:** Tally Cohen, Jeanna Starosvetsky, Uta Cheruti, Robert Armon

**Affiliations:** Faculty of Civil & Environmental Engineering, Division of Environmental, Water and Agricultural Engineering, Technion, Haifa 32000, Israel; E-Mails: tallyco@tx.technion.ac.il (T.C.); starjean@tx.technion.ac.il (J.S.); uta@tx.technion.ac.il (U.C.)

**Keywords:** sol-gel, bacteria, protozoan parasite, whole cell imprinting, biosensors, sol-gel films

## Abstract

Thin films of organically modified silica (ORMOSILS) produced by a sol-gel method were imprinted with whole cells of a variety of microorganisms in order to develop an easy and specific probe to concentrate and specifically identify these microorganisms in liquids (e.g., water). Microorganisms with various morphology and outer surface components were imprinted into thin sol-gel films. Adsorption of target microorganism onto imprinted films was facilitated by these macromolecular fingerprints as revealed by various microscopical examinations (SEM, AFM, HSEM and CLSM). The imprinted films showed high selectivity toward each of test microorganisms with high adsorption affinity making them excellent candidates for rapid detection of microorganisms from liquids.

## 1. Introduction

In the last decades sol-gel (SG) chemistry has played a major role in the evolution of hybrid materials through bridging organic and inorganic chemistry. It is considered one of the fastest growing fields of contemporary chemistry [1–5]. Organically modified silicate materials (ORMOSILS) involved in sol-gel processes under ambient conditions, are a highly attractive research area due to their simplicity and versatility. Sol-gel technology combining composition and microstructure control at a molecular level yields the ability to shape these materials in various configurations: bulk, thin films, fibers, monoliths and powders. Reagents/reactants can be readily incorporated in a stable host matrix by simply adding it to a sol precursor prior to its gelation at room temperature. In some cases, the matrix may even stabilize the entrapped reagent from photodegradation and other extreme environmental conditions [6,7]. Therefore, SG materials are chemically, photochemically and electrochemically stable [4]. The attractiveness of these materials can be attributed to three main factors: (1) the ability to generate an almost infinite number of such hybrid materials that demonstrate both the mechanical stability of an inorganic framework and the particular reactivity (e.g., selective recognition, optical properties, electrochemical activity, *etc.*) of the organic components; (2) SG-derived materials can be used to encapsulate bio-molecules (e.g., enzymes, antibodies and other proteins) in a functional state without structural damage; (3) discovery of the supra-molecular template approach which can generate ordered microstructures over long length scales [5].

At present SG technology is practically applied in all known branches of modern industry: advanced medical technologies, electronics, analytical chemistry, biochemistry, electrochemistry, catalysis, optics, development of new composite materials, imprinting, *etc.* [2,5–7].

Among the many materials produced by the SG technique, sensing devices are a major area [7–9]. High sensitivity, good selectivity, long term durability (which also implies good reproducibility and reversibility) ease of modification and flexible processing are key parameters for successful applications of this methodology in biosensor construction and development [10–14]. The introduction of molecularly imprinted polymers (MIP) in sensor applications opened a most promising field with industrial implementations [15–17]. At present molecular imprinting (MI) is a well established method for designing highly selective sensors [18]. According to this technique, a polymeric network is assembled and molded around a suitable template molecule, which upon removal, yields micro-cavities with a specific size, shape, and/or chemical functionality in a highly cross-linked matrix. Such molecularly designed cavities show affinity for the imprinted molecule over other structurally and chemically related compounds. Preparation of thin films by this method is very attractive for chemical and biological sensing applications, since the reduced response time for the reagent due to significant diffusional path length shortening. Another feature that may improve diffusional penetration into the polymeric matrix is its porosity. SG materials inherently combine these two requirements, namely the ability to form very thin films and variable matrix porosity. In addition, the ease of SG fabrication, mild reaction conditions, commercial availability of a wide variety of functional monomers, physical rigidity of the matrix, chemical inertness, and resistance to thermal and solvent stresses, had all made the SG methodology attractive for molecular imprinting of thin films and a perfect basis for designing various biological applications [4–7].

During the last decades, the scope of analytical chemistry has shifted from simple molecules to increasingly complex organic and biological systems (e.g., proteins, carbohydrates, lipids and nucleic acids) and even more towards entire biological species, such as eukaryotic, bacteria and viruses cells [10–13,19–21]. For these analytes (in the present study, bacterial cells) an increasing demand for fast, high value and on-line analysis has developed. Development of such detection methods is essential in many areas like medicine (infectious diseases), food industry, water supply and environmental microbiology. The most common chemical and biological methods relay on sample inoculation of a specific nutrient media (solid or liquid) followed by incubation at a certain temperature allowing microorganisms to grow and form colonies. The newly developed colonies are counted and identified based on biochemical tests or genotypically by polymerase chain reaction (PCR) and electrophoresis [22]. These methods are suitable for this task and yield the desired detection limits with high specificity, however they are still too long in time terms for the rapid determinations required by several areas such as medicine and security. For example, bacterial cultivations on specific media may require many hours or even days. Recently, a large effort has been made to overcome this problem when dealing with environmental samples that beside complex composition harbor few bacteria therefore requiring a concentration step. Many sophisticated methods for detection and identification of specific microorganisms were developed, however little effort had been devoted to develop methods for selective capture and concentration of microorganisms at species level from the environment. Such methods would play a critical topmost role as a capture/concentrator/identifier for biological warfare agents or medical/environmental pathogens of concern. Various sensors for microorganisms detection, were proposed based on different approaches using various bioactive materials like antibodies, proteins, cell’s membrane components, *etc.* [8–12,22]. Although microorganism’s sensors provide critical screening capability as early warning systems, they generally lack specificity toward specific microorganisms. An obvious requirement exists for a rapid, selective seizure and detection of microorganisms from highly complex environments.

The new emerging approach for selective organism capture based on sol-gel bio-imprinting was firstly demonstrated by Dickert *et al.* [23,24] that developed a soft, lithographic technique used to produce an imprinted surface on a quartz crystal microbalance (QCM) sensor. The imprinted layer was capable of selective capture of different yeast genera. Cunliffe *et al.* [25], through a complex multi-step organic synthesis, prepared bacterially imprinted polymeric surfaces favoring attachment of affinity ligands solely onto an imprinted site. These unique imprinted materials are capable of microorganism trapping by combined size-shape discrimination and affinity recognition. Perez *et al.* [26] demonstrated selective binding of rod-shaped *(Listeria monocytogenes*) and coccoidal (*Staphylococcus aureus*) bacteria from a mixture of both on beads imprinted with one or the other. Harvey *et al.* [27] used affinity augmented beads imprinted with *Bacillus thuringiensis* and *Bacillus anthracis* as a semi-selective matrix to capture and concentrate their specific spores based on same principles.

In our laboratory, previous investigations revealed a variety of successful applications of ormosil sol-gel processes in environmental biotechnology such as: entrapped *Thiobacillus thiooxidans* free cells extract in a SG matrix effectively oxidized H_2_S in water [28]; epifluorescent microscopy detection of *E. coli* esterase/lipase activity by thin SG films doped with fluorescein diacetate [29]; SG entrapment of *Pseudomonas spp*. parathion hydrolase catalyzing hydrolysis of organophosphates [30], effective reduction of nitrate/nitrite in solution by up-flow sludge blanket denitrifiers whole cell entrapped in SG matrix [31] immobilization of humic acids in SG matrix and investigation of adsorption kinetics of hydrophobic contaminants to soil [32] and tissue culture growth on sol-gel thin layers for rapid detection of viral plaques [33,34].

The aim of the present study was to prepare and evaluate SG imprinted surfaces (ormosils-based) with various microorganisms to efficiently discriminate between different mixed microorganisms in liquid environment (e.g., water) based on their morphology or outer layer components regardless of their similar morphology. The basic idea was to perform whole cell imprinting in SG precursor materials (tetraethyl orthosilicate-TEOS, tetramethyl orthosilicate-TMOS, *etc.*) in order to obtain bacterially imprinted thin films (1–2 μm thick) that after immersion for a short time in a liquid volume will selectively adsorb planktonic cells into already formed micro-cavities.

## 2. Results and Discussion

### 2.1. Imprinting of SG Films with Whole Cell Bacteria

**Figure 1.**
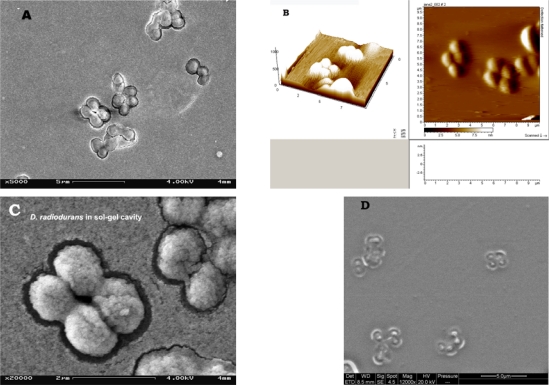
Images of imprinted *D. radiodurans*: entrapped in SG-film (SEM) **(A)**, AFM **(B)**, HSEM **(C)** and SG-film cavities left after removal of entrapped bacterial cells (SEM) **(D)**.

The extremely radioresistant Gram positive bacterium *Deinococcus radiodurans* strain UWO 288 was selected as template for sol-gel imprinting due to its coccoidal cells organized in pairs and tetrads [35]. Beside radioresistance, *D. radiodurans* has another interesting feature relevant to the imprinting process, namely its outer membrane structure containing a regular surface protein array (RS) or so-called hexagonally packed intermediate layer (HPI) [42,44]. According to Thompson and Murray [43] blebs from the outer membrane of this bacterium are shed as large vesicles from approximately 5% of the cell population during growth. Elution of entrapped *D. radiodurans* cells into thin SG films (∼1 μm), casted on microscope slides, revealed paired/tetrad like cavities similar to *D. radiodurans* cells morphology (Figure 1). Figure 1 (A, B and C) shows the morphology of *D. radiodurans* cells entrapped into sol-gel film observed with different microscopes. Interestingly, it was found that sole Gram staining procedure is enough to cause elution of the imprinted cells (at the moment under study to comprehend this phenomenon) leaving clear paired or tetrad cavities as observed with SEM (Figure 1D).

**Figure 2.**
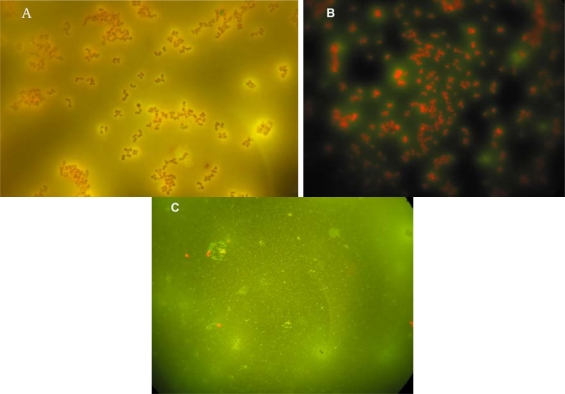
*D. radiodurans* specific adsorption onto SG imprinted films as detected by acridine orange staining by epifluorescent microscopy (×1,000). **(A)** *D. radiodurans* imprinted SG film(Gram staining and AO); **(B)** imprinted SG film with *D. radiodurans* exposed to new planktonic suspension of *D. radiodurans* (AO staining); **(C)** Control, non-imprinted sol-gel film exposed to same *D. radiodurans* suspension (AO staining).

Sol-gel imprinted films were also subjected to acridine orange (AO) staining to be used with epifluorescent microscopy (Figure 2). AO staining of cavities formed following elution of imprinted *D. radiodurans* revealed staining of residual components entrapped in a sol-gel layer (Figure 2A). As already mentioned earlier, these components are apparently membrane blebs shed by this bacterium during the imprinting process. A similar phenomenon was observed with other bacteria such as *Escherichia coli* CN_13_, *Sphaerotilus natans* and *Bacillus subtilis* (data not shown). In order to test the affinity of *D. radiodurans* planktonic cells in suspension towards imprinted SG film, an imprinted probe was immersed into a fresh *D. radiodurans* suspension (Figure 2B) against a non-imprinted sol-gel film as control (Figure 2C). The imprinted SG film was selectively covered with adsorbed *D. radiodurans* only onto imprinted sites compared with control that showed only few sparse non-specific adsorbed cells (Figure 2C).

**Figure 3.**
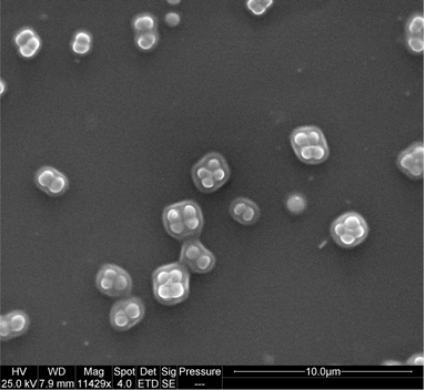
SEM micrograph of SG film imprinted with *D. radiodurans* cells and exposed to fresh *D. radiodurans* suspension for 30 min.

The imprinting specificity for *D. radiodurans* cells was further substantiated by SEM micrographs (Figure 3). Imprinted SG film exposed to fresh *D. radiodurans* suspension clearly revealed adsorption and location of these bacteria pairs/tetrads solely in formed cavities.

**Figure 4.**
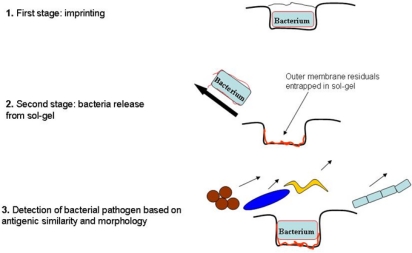
Schematic representation of whole cell imprinting of ormosils thin films through a SG procedure.

As already mentioned, it was assumed that following the imprinting procedure a number of membrane surface components of entrapped/imprinted bacteria are left in the sol-gel matrix. In our opinion, these molecular components facilitate adsorption/attachment of free cells in suspension onto imprinted SG films. A schematic potential explanation of this process is presented in Figure 4. Briefly, a morphological defined bacterium (e.g., rod shaped) is entrapped into a sol-gel thin film (approx. 1–1.5 μm) partially or completely (Figure 4, first stage). Some of the SEM micrographs revealed bacterial cells dipped into the SG layer and covered with SG material as a very thin cap, while others were just sunk without any cover layer. Release of entrapped bacteria revealed, beside cell morphology, certain membrane components left entrapped into the SG formed cavity (Figure 4, second stage). It should be mentioned, that SG cover of entrapped bacteria during imprinting process is very fragile and it is easily removed by elution procedure leaving a clear morphological imprint. Based on these characteristics, target microorganisms are easily adsorbed onto an imprinted probe for recognition and further analysis, while discriminating between various morphologies and cell surface components (Figure 4, stage 3).

### 2.2. Analysis of SG Entrapped Membrane Residuals

**Figure 5.**
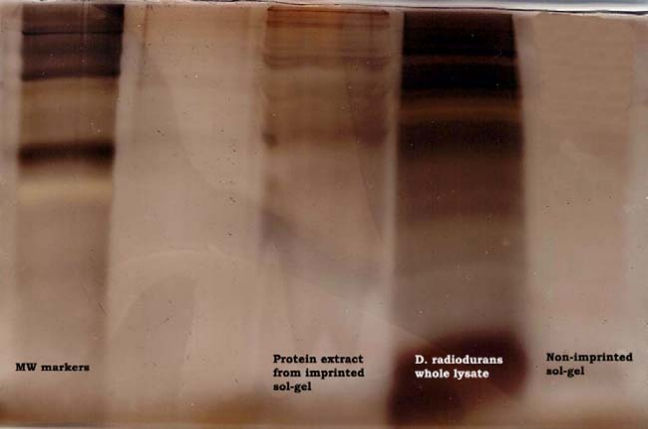
Silver stained SDS-PAGE gel of SG films imprinted with *D. radiodurans* and non-imprinted. From left to right: first column: molecular markers; third column: imprinted SG with *D. radiodurans*; fourth column: *D. radiodurans* whole cell lysate; fifth column: non-imprinted SG film.

*D. radiodurans* residual bacterial surface components left in SG films were further analyzed electrophoretically for the presence of protein/glycoproteins against non-imprinted SG film and whole cell lysate (Figure 5).

Controls were: whole cell (diluted to the equivalent total protein concentration as imprinted sample), and a negative control of non-imprinted dissolved SG films. The imprinted SG films revealed protein bands corresponding to whole cell lysate, but at lower intensity. As expected, no protein bands were seen with non-imprinted SG films. These quantitative results, clearly confirmed the previous microscopic observations (Figure 2) that residual membrane proteins had been entrapped in the SG film imprinted cavities.

### 2.3. Imprinting of SG-films with Other Microorganisms

**Figure 6.**
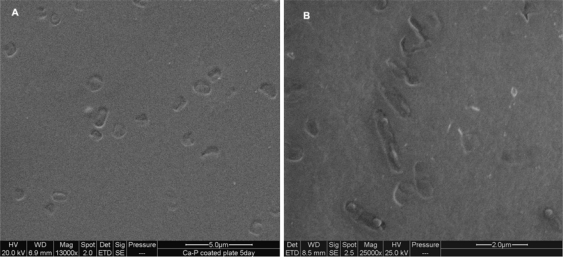
SEM micrograph of imprinted SG film with *E. coli CN_13_* **(A)** and **(B)** after 30 minutes exposure to new *E. coli CN_13_* suspension.

**Figure 7.**
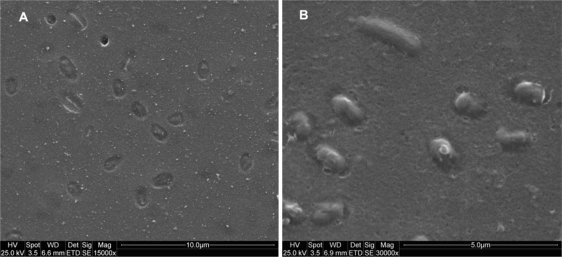
SEM micrograph of imprinted SG film with *B. subtilis* **(A)** and **(B)** after 30 minutes exposure to new *B. subtilis* suspension.

**Figure 8.**
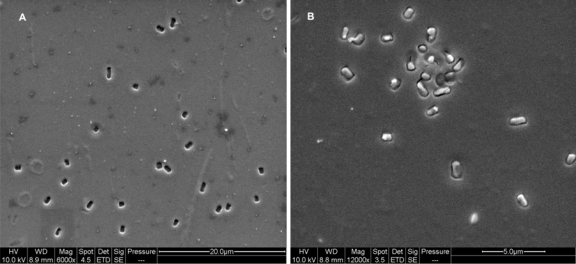
SEM micrograph of imprinted SG film with *S. natans* **(A)** and **(B)** after 30 minutes exposure to new *S. natans* suspension (B).

**Figure 9.**
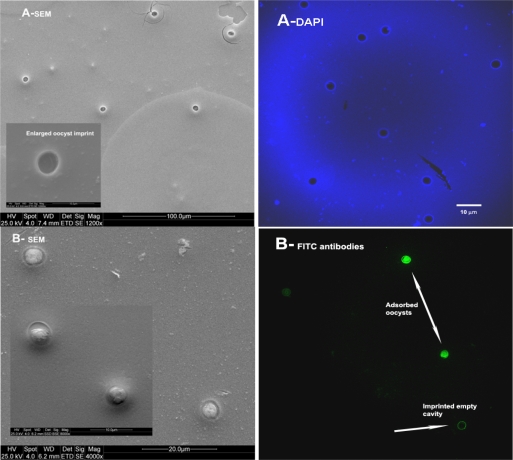
Images of imprinted SG films with *Criptosporidium parvum* oosysts and adsorption of new oocysts in suspension. **(A)** SG imprinted film (left-viewed with SEM; right-viewed with CLSM) and **(B)** SG imprinted film exposed for 30 minutes to new oocysts suspension (left-viewed with SEM; right viewed with an epifluorescent microscope).

In order to assess the versatility of whole cell imprinting method in sol-gel films, bacteria such as *E. coli CN_13_*, *S. natans*, *B. subtilis* and oocysts of the protozoan parasite *Cryptosporidium parvum* were used as templates (Figures 6–9). In all imprinted SG films, a well defined morphology of these microorganism species was visible, including molecular fingerprints left by imprinted cells. This characteristic can be clearly seen in Figure 9B, where *Cryptosporidium parvum* oocysts were stained with FITC antibodies and examined with an epifluorescent microscope. As these antibodies are specific to oocysts’ outer layers, empty imprinted cavities were stained only circumferentially revealing outer layer residuals entrapped into SG film. Exposure of imprinted SG films revealed excellent reinclusion of planktonic cells in suspension only onto formed cavities without any nonselective adsorption on non-imprinted sites. Non-imprinted SG control films revealed extremely low adsorption (close to zero) of the different microorganisms that may be explained by elevated hydrophobicity of prepared films. In spite of SG-films hydrophobicity, subsequent to imprinting process, cells adsorption ability increased dramatically providing the evidence that template cavities changed their surface chemistry due to residual outer layer entrapment during the process as already shown [12].

An interesting imprinting feature was observed with rod shaped bacteria, in contrast with spherical ones (e.g., *D. radiodurans*). Geometrically obvious, spheres (cocci) entrapped into SG films will form the same cavity shape no matter the orientation of their deposition, however rod shaped bacteria can form two shaped cavities: rectangular and square, depending on SG film deposition of rod bacteria (see Figure 8A). However, new planktonic bacteria reinclusion revealed correct redirection of rods according to imprinted outline (Figure 8B).

### 2.4. Sensitivity and Selectivity of Imprinted SG-films

**Figure 10.**
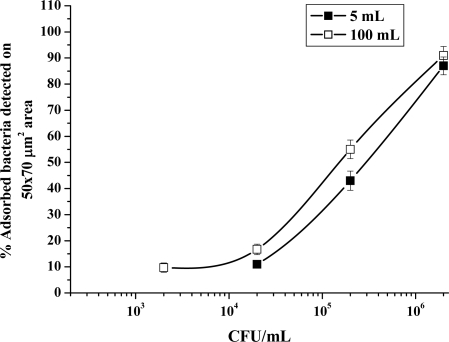
Sensitivity of imprinted SG films to reinclude *D. radiodurans* and *E. coli* cells (separately) in two different volumes (5 and 100 mL).

To test sensitivity, adsorption of bacteria in suspension (*D. radiodurans* and *E. coli* CN_13_ separately) onto certain imprinted SG film area, was measured (Figure 10). At 10^3^ to 10^4^ CFU/mL a 10% recovery of these bacteria was observed which seems to be the threshold of this method sensitivity. Increasing target planktonic bacteria numbers improved adsorption onto SG films to approx. 90%. Adsorption volume of 100 mL was slightly more sensitive compared to 5 mL. Future studies will be performed in order to increase sensitivity through better contact between planktonic cells and imprinted film.

To determine selectivity, two experiments were carried out: (1) **Differentiation between different bacterial morphologies**-in which *D. radiodurans* SG imprinted films were exposed to a mixture of *D. radiodurans, E. coli CN13* and *B. subtilis* in suspension (10^8^ CFU/mL each) for 30 minutes. The SG film screened under SEM and epifluorescent microscope revealed sole adsorption of *D. radiodurans* cells onto the formed cavities (data not shown); (2) **Differentiation between same bacterial morphologies**-in which *E. coli* CN_13_ SG imprinted film was exposed to *B. subtilis* in suspension (10^8^ CFU/mL) for 30 minutes. Both bacteria have a rod like morphology with a size difference (*B. subtilis* is larger in comparison with *E. coli*). The exposed SG films revealed adsorption of *E. coli* CN_13_ cells only, as observed by specific fluorescent antibodies staining with no adsorption onto non-imprinted control SG films (data not shown). *B. subtilis* imprinted films exposed to *E. coli* did not reveal any adsorption of the last (data not shown).

## 3. Experimental Section

### 3.1. Experimental Microorganisms and Growth Conditions

Bacteria such as *D. radiodurans*, *S. natans, E. coli CN_13_*, *B. subtilis* and oocysts of protozoan parasite *Cryptosporidium parvum* were used as imprinting templates due to their morphology and size variations.

*D. radiodurans* UWO 288 bacterium (ATCC13939) (kindly donated by Prof. R.G. Murray, University of Western Ontario, Canada) has a morphology of spherical cells (Ø = 0.5–3.5 μm) organized in pairs or tetrads. *D. radiodurans* was grown in TGY medium at 36 °C for 36–48 hours as already described [35].

*S. natans* bacterium (from our collection, primarily isolated from corrosion deposits of a cooling water heat exchanger at Haifa’s Refinery Plant) is a Gram negative (G-) rod filamentous iron oxidizing bacteria with a cell length from 2 to 12 μm, depending on growth conditions. *Sphaerotilus natans* was grown in Winogradsky medium containing (g/L): NH_4_NO_3_ (0.5), NaNO_3_ (5), 0.66 K_2_HPO_4_·3H_2_O (0.66), MgSO_4_ (5), CaCl_2_ (0.1), ferric ammonium citrate (10) and incubated at 28 °C for 4–5 days. Under these conditions, bacterium length was 2–2.5 μm.

*E. coli CN_13_* (a nalidixic acid resistant G- *E. coli* C strain) [36] and *B. subtilis* (G+) (Difco,USA) with cell sizes of 0.5 × 1.5 μm and 0.7 × 3 μm, respectively, are both rod-shaped bacteria selected in order to assess selectiveness towards different bacterial species with similar morphology. *E. coli* CN_13_ was grown on R_2_A agar (Acumedia, USA) at 37 °C for 48 hours and *B. subtilis* in nutrient broth (Difco, USA), both shaken (50 rpm) at 30 °C for 24 hours. All experimental bacterial species stocks contained 10^7^ to 10^9^ CFU/mL.

Protozoan parasite *Cryptosporidium parvum* oocysts with spherical pea-like morphology and an average size of 5.5 μm were purchased from Waterborne Co. (New Orleans, USA). Stock oocysts, (10^8^/mL) were washed three time with sterile saline before use.

### 3.2. Imprinted Sol-Gel Films Preparation

#### 3.2.1. Stock of Sol-Gel Solution

Tetraethoxysilane (TEOS, 98%, ABCR Co., Germany) precursor was used as bulk sol-gel solution. TEOS (2 mL) was mixed with triple-distilled water (1 mL) and gently stirred for 10 min at room temperature (22 ± 1 °C). Next, 0.1 M HCl (0.1 N, 0.25 mL) was added to the solution which was further stirred for 3–5 hours until visible homogeneity. The final solution was aged at 4 °C for 24 hours and kept for approx. 5 weeks at 4 °C.

#### 3.2.2. Template cells preparation

For the imprinting procedure, bacterial cells were harvested either by scraping plates from Petri dish surfaces with a sterile loop when grown on solid media or by centrifugation (6,000 × g, 10 min) when grown in liquid medium. Harvested cells were washed twice with sterile saline solution (0.85% NaCl) and then re-suspended by vortex for 7 minutes in 30 mL rinsing solution supplemented with sterile Tween 80 (0.2%) to reach 10^9^–10^10^ CFU/mL. Tween 80 was added in order to disperse cell aggregates. The bacterial suspension was further vortexed for additional 10 minutes to reach homogeneity. *Cryptosporidium* parvum oocysts for imprinting were prepared as follows: 1mL of stock solution was centrifuged (1,700 × g) for 10 minutes and the pellet resuspended in sterile saline supplemented with Tween 80 (0.2%). The procedure was repeated three times to remove any present preservatives.

### 3.3. Imprinting Procedure

Sol-gel films (SG) were prepared on standard microscope glass slides previously cleaned according to a modified procedure [16]. Briefly, glasses were soaked for 20–30 min successively in 1:1:1 acetone-ethanol-chloroform mixture; soap solution; Piranha solution—H_2_SO_4_:H_2_O_2_ (4:1), then thoroughly rinsed in double-distilled water followed by drying at 100 °C for 2–4 hours.

The imprinting procedure was as follows: aged sol-gel stock solution (0.5 mL) was added to bacterial suspension (3 mL, 10^9^ to 10^10^ CFU/mL) and stirred for 8 to 10 minutes. Subsequently a glass slide was drop-coated with 0.25 mL of this experimental mixture. In order to obtain a thin and uniform film, the glass slide was instantly positioned vertically to drain excess solution. With *Cryptosporidium parvum* oocysts, 1 mL suspension (1.2 × 10^7^ oocysts) was mixed with 166.7 μL of the sol-gel stock solution stirred for the same time interval and slide spread. Slides coated with sol-gel films containing bacteria/oocysts were placed into a desiccator and dried over night.

Removal of immobilized bacterial cells from dried sol-gel film was performed by soaking the coated slides for 30–40 min in 96% ethanol and washed with sterile double-distilled water. Slides were further Gram stained (successively 3 min in crystal violet, iodine, 70% ethanol, safranin for 3 minutes each) (Bactolab Diagnostics, Israel) to visually detect cavities formed by entrapped bacteria. It is important to note that in many cases immobilized bacterial cells dropped just after Gram staining procedure without ethanol treatment. Removal of *Cryptosporidium* oocysts was done by exposure of sol-gel coated slides to a NaCl solution (10% w/v) for 4 hours. Control glass slides were coated with sol-gel films without bacteria (as non-imprinted films) and rinsed with suspension media (0.85% NaCl + 0.2% Tween 80).

### 3.4. Detection of Sensitivity and Selectivity Ability of Imprinted SG-films

Readsorption/reinclusion tests were carried out with SG film coated slides (imprinted and non-imprinted) by exposure to 10^7^–10^9^ CFU/mL of an appropriate bacterial suspension (5 and 100 mL volume) for 30 min with gentle stirring. Following exposure, slides were gently washed with sterile triple-distilled water and then examined under an epifluorescent microscope (×1,000, Zeiss Axiolab, filters 450–490 excitation and FT510-LP520 emission), confocal laser scanning microscope (CLSM) (DM5500 Q, Leica, Germany) at ×630 magnification, SEM (LEO-982 Geminate FEG-HRSEM, Germany and a dual-beam focused ion beam FIB, FEI Strata 400-S, USA) and AFM (AFM/STM, PicoScan, Molecular Imaging, USA). Slides for epifluorescence microscopy were previously immersed in a glutaraldehyde solution (2%) for 10 minutes and subsequently for additional 10 minutes into acridine orange (AO) solution (0.01% AO in 0.1 M phosphate buffer, pH 7.0, w/v) before observation. SEM samples were dehydrated and critical-point dried, fixed on aluminum stubs, sputter coated with gold-palladium and examined with a scanning electron as already described [37]. AFM samples were performed at 20 °C in phosphate buffer (10mM, pH 7) KH_2_PO_4_ and contact mode images were taken in constant force mode with applied force of ∼1 nN, and scan rate between 1 and 2.5 Hz [38].

*Cryptosporidium parvum* oocyst enumeration in suspension was determined by monoclonal antibodies staining and epifluorescence. Briefly, 500 μL of oocyst suspension was placed in an Eppendorf standard microtest tube (2 mL volume). To this suspension, a volume of 250 μL fluorescein isothiocyanate (FITC)-conjugated monoclonal antibody (Crypto-A-Glo Kit, Waterborne Co., USA) was added and the mixture was incubated for 30 min at 36 °C. Following incubation, the stained sample was subjected to three rinses with phosphate buffered saline (pH 7.0, 0.1 mM) and centrifugally washed (4,000 × g) for removal of unattached antibodies. Then, 100-μL aliquots were placed on six-well chamber slides and fixed with 100% methanol. After methanol vaporization at 36 °C, each well containing stained oocyst sample was covered with 35 μL of mounting medium (2%) (1,4-diazabicyclo-2.2.2-octane, DABCO, Aldrich, USA) in glycerol and a coverslip. The entire well was scanned with an epifluorescence microscope (AxioLab, Zeiss) at ×1,000 magnification. Imprinted and reincluded oocysts were directly stained with FITC-conjugated monoclonal antibody, rinsed with phosphate buffer (0.01 M, pH7), dried and observed with epifluorescent microscope as mentioned above. Pea-like oval particles with suture on its envelope, 4 to 6 μm in diameter and with brilliant apple-green fluorescence glow, were identified as *C. parvum* oocysts.

### 3.5. Analysis of Outer Cell Components Left in Sol-Gel Cavities Following Templates Removal

#### 3.5.1. Bacteria Lysates

*D. radiodurans* cells were washed once in cold phosphate buffer saline (PBS, 0.1 mM, pH 7.0) and resuspended in 5 mL of the same cold buffer containing 200 μg/mL of freshly made lysozyme (Sigma, USA) and incubated for additional 30 minutes on ice to obtain spheroplasts. Spheroplasts were supplemented with protease inhibitor cocktail Set 1 (500 μM AEBSF, 150 nM aprotinin, 1 μM E-64 protease inhibitor, 0.5 mM EDTA and 1 μM leupeptin) (Calbiochem, Israel)) and sonicated for 4 minutes (40% amplitude, cycle time 0.5 seconds) on ice. The admixture was centrifuged (11,000 × g) for 15 minutes. The supernatant was collected and separated from the pellet.

#### 3.5.2. Membrane Components Extraction from Sol-Gel

Thirty microscope slides were imprinted with *D. radiodurans* pure culture cells. Imprinted cells were removed from the SG films by Gram staining as already described above. The cavitated slides were then immersed in NaOH (0.5M) for 48 h in order to dissolve sol-gel layer and finally scraped with a sterile cell scraper. The final solution was dialyzed over night against Tris-HCl buffer (20 mM, pH 6.8) and further concentrated by polyethylene glycol flakes (PEG 6000) up to 1 mL final volume. The remaining concentrated solution was centrifuged (11,000 × g) for 15 minutes and supernatant separated from pellet. The final pellet was resuspended in 1% NP-40 lyses buffer (10 mM Tris pH 7.5, 150 mM NaCl, 1% NP-40, 5 mM EDTA, 10% glycerol) and total protein was determined by a Bradford assay [39].

#### 3.5.3. Membrane Proteins/Glycoproteins SDS-PAGE Determination

Briefly, electrophoretic gels were prepared in Mini-Protean Tetra-Cell (Bio-Rad, USA), with separating gel (10%) and stacking gel (4%) as already described [40]. Gels were stained by silver nitrate (0.1%) as described [41].

## 4. Conclusions

Diaz-Garcia and Laiño, in a comprehensive review, outlined the recent developments and applications of imprinted sol-gel materials [47]. Main progress in imprinting was achieved in the field of chemical compound detection and only a few reports dealing with yeast whole cells were mentioned. Since then, other microorganisms such as tobacco mosaic virus (TMV) [46] and *E. coli* [23] were also imprinted in a monomer mixture of styrene, divinylbenzene, methacrylic acid and TEOS sol-gel respectively. Dickert and Hayden [24] showed that the yeast *S. cereviseae* imprinted in polyurethane has the potential to reinclude these microorganisms under flow conditions with high selectivity and sensitivity [45]. Detection of whole yeast cells imprinted on films made of polyurethane and alkoxide through sol-gel technique was done by QCM [24]. Comparison of both imprinting materials showed that alkoxide sol-gel layers were more robust, though only minor sensor responses were observed with tested yeast on alkoxide imprints. The observed sensitivity on polyurethane was between 10^4^ to 10^6^ CFU/mL. In the present study, it has been shown that ormosil (TEOS) imprinted with various bacterial species resulted in highly specific cell reinclusion following exposure to planktonic cells in water suspensions. The high affinity of imprinted sol-gel films toward planktonic cells was found to be determined by cavity morphology (rod, coccus, tetrad) and residual components if the microorganism’s outer surface was entrapped into the cavity surface area. These two characteristics were found to be specific enough to discriminate between the various bacteria tested without interference between the different species when mixed together. Broadening this approach to oocysts (the excreted form) of the protozoan parasite *Cryptosporidium parvum*, resulted in similar excellent results. The alkoxide sol-gel method performed at room temperature by very simple chemical steps is an excellent method for molecular imprinting and recognition of whole cells. Furthermore, during the gelation process microorganism outer surface components were entrapped (mechanical peeling), providing an affinity factor that specifically enhanced reinclusion. Further studies will concentrate on fine tuning of the present method to lower detection level and improving QCM application for faster results, as required in some circumstances. In addition, bacteria with similar morphology and size such as *E. coli*, *Salmonella thyphimurium*, *Citrobacter freundii* and *Erwinia carotovora* (that belong to the Enterobacteriaceae family) will be imprinted and cross-exposed to further verify specificity.
